# Case Report: Osmotic Demyelination Syndrome After Transcatheter Aortic Valve Replacement: Case Report and Review of Current Literature

**DOI:** 10.3389/fmed.2022.915981

**Published:** 2022-06-20

**Authors:** Xinhao Jin, Yonggang Wang

**Affiliations:** Department of Critical Care Medicine, Sir Run Run Shaw Hospital, Zhejiang University School of Medicine, Hangzhou, China

**Keywords:** osmotic demyelination syndrome (ODS), transcatheter aortic valve replacement, case report, hyponatremia, serum sodium

## Abstract

**Background:**

Osmotic demyelination syndrome (ODS) has a low incidence but is a life-threatening neurological disorder whose common cause is rapid overcorrection of chronic hyponatremia. Transcatheter aortic valve replacement (TAVR) is a new and important therapy for patients with aortic valve stenosis. In this article, we discuss the case of a 64-year-old woman who developed ODS after TAVR and provide a literature review.

**Case Presentation:**

A 64-year-old female patient was admitted to the hospital with chest tightness, shortness of breath, and fatigue for 2 months, with worsening of symptoms for 3 days prior to presentation. Auscultation revealed crackles in the lung fields, and systolic murmurs could be easily heard in the aortic area. Echocardiography showed severe aortic stenosis. Chest X-ray showed pulmonary oedema. Laboratory examinations showed that her serum sodium was 135 mmol/L. The patient received a diuretic to relieve her symptoms but showed little benefit. Her symptoms worsened, and her blood pressure dropped. Then, she underwent emergency TAVR under extracorporeal membrane oxygenation (ECMO) support. After the operation, her urine output increased markedly, and serum sodium increased sharply from 140 to 172 mmol/L. An MRI scan showed multiple lesions in the pons suggestive of ODS.

**Conclusion:**

To date, this is the first reported case of a patient who developed ODS after receiving TAVR. In current clinical practice, diuretics are often used in aortic stenosis patients because of pulmonary oedema. After a patient receives TAVR, kidney perfusion pressure quickly returns to normal, and with the residual effect of a high-dose diuretic, balances of fluid volume and electrolyte levels in this phase are quite fragile and must be carefully managed. If a patient has neurological symptoms/signs during this phase, ODS should be considered, and MRI might be necessary.

## Background

Osmotic demyelination syndrome (ODS), which includes central pontine myelinolysis (CPM) and extrapontine myelinolysis (EPM), is defined as degeneration of myelin within the central nervous system with sharply demarcated lesions within the brain, especially the pons, and is diagnosed by magnetic resonance imaging (MRI) (1). ODS accounts for ~0.4–0.56% of all neurological admissions to tertiary referral hospitals, and MRI-based studies describe an incidence of ODS ranging from 0.3 to 1.1% (2). Although ODS is a low-incidence disorder, it is associated with high rates of disability and mortality (3). Most published cases are related to rapid correction of hyponatremia in patients with chronic hyponatremia.

Aortic stenosis (AS) has estimated prevalence rates of 12 to 13% for all AS cases and 2 to 4% for severe AS cases in patients ≥75 years of age in the Western world (4). Severe, symptomatic aortic stenosis is fatal; if left untreated, the mortality rate is 50% at 2 years (5). In 2002, the first human transcatheter aortic valve replacement (TAVR) was performed on a 57-year-old man in Rouen (France) (6), which introduced a new interventional era. TAVR is a relatively recent revolutionary treatment that has grown exponentially over the past decade and has become the mainstay of treatment for symptomatic severe aortic stenosis.

In this article, we report a case of ODS that occurred in a patient with aortic stenosis after TAVR under extracorporeal membrane oxygenation (ECMO) support. A diuretic phase was induced by a sudden improvement of low kidney blood perfusion pressure after TAVR. Balances of fluid volume and electrolyte levels in this phase are quite fragile and must be carefully managed.

## Case Description

**Figure 1:**
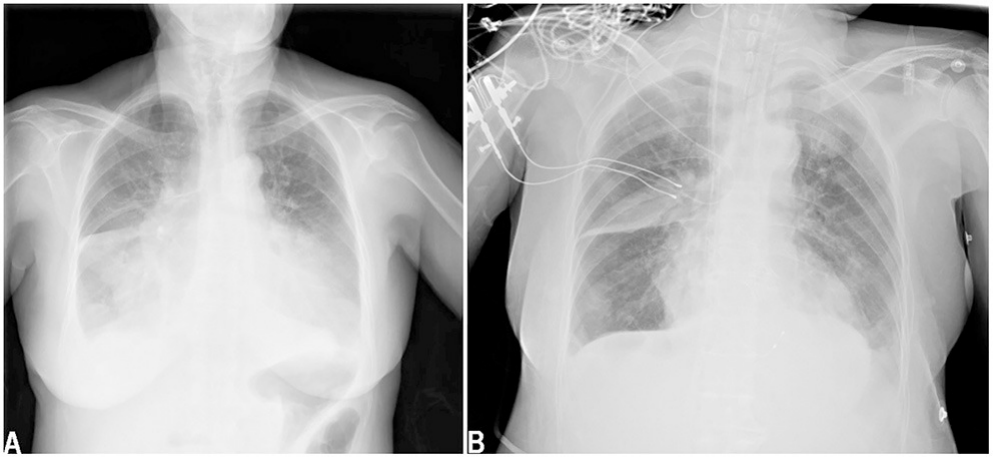
Chest x-ray showed pulmonary oedema and bilateral pleural effusion when admission **(A)**. Chest x-ray showed pulmonary oedema and bilateral pleural effusion decreased after TAVR **(B)**.

A 64-year-old female patient (140 cm/48.6 kg, BMI: 24.8) presented to the hospital with chest tightness, shortness of breath, and fatigue for 2 months, with symptom worsening for 3 days prior to presentation. The patient had no history of shortness of breath or rapid breathing. Her past surgical history included cholecystectomy 10 years prior. She was not epileptic, diabetic, or hypertensive and never smoked or drank alcohol. She and her family members had no history of kidney diseases. On admission, she had a temperature of 36.3°C, a pulse of 97 beats per minute, a blood pressure of 96/65 mmHg, and a respiratory rate of 24 cycles per minute. Her consciousness was clear, and her Glasgow Coma Scale score was 4+5+6. Auscultation revealed crackles in the lung fields, the cardiac rhythm was regular, and a systolic murmur could be easily heard in the aortic area. Both lower extremities showed mild pitting oedema. Echocardiography showed severe aortic stenosis: the aortic valve area (AVA) was 0.28 cm^2^, and the ejection fraction (EF) was 23%. Color Doppler flow imaging (CDFI) showed that the blood flow of the aortic valve increased significantly, the peak velocity was 5.7 m/s, the peak gradient was 130 mmHg, and the mean gradient was 81 mmHg. The structures of the bicuspid valve and tricuspid valve were normal, but moderate regurgitation was evident in both the bicuspid and tricuspid valves. Chest X-ray showed pulmonary oedema and bilateral pleural effusion (Figure 1A). Lab examinations showed that her serum sodium was 135 mmol/L, AST was 396 U/L, ALT was 440 U/L, proBNP was >25,000 pg/mL, troponin I was 0.120 ng/mL, and lactic acid was 5.3 mmol/L. The patient's glomerular filtration rate was 50.5 ml/min, BUN was 12.82 mmol/L, creatinine was 76 μmol/L, serum osmolarity was 296 osmo/kg, urine osmolarity was 490 osmo/kg, urinary specific gravity was 1.014, and urine output was 950 ml/24 h. Urine glucose and urinary protein were negative.

**Figure 2:**
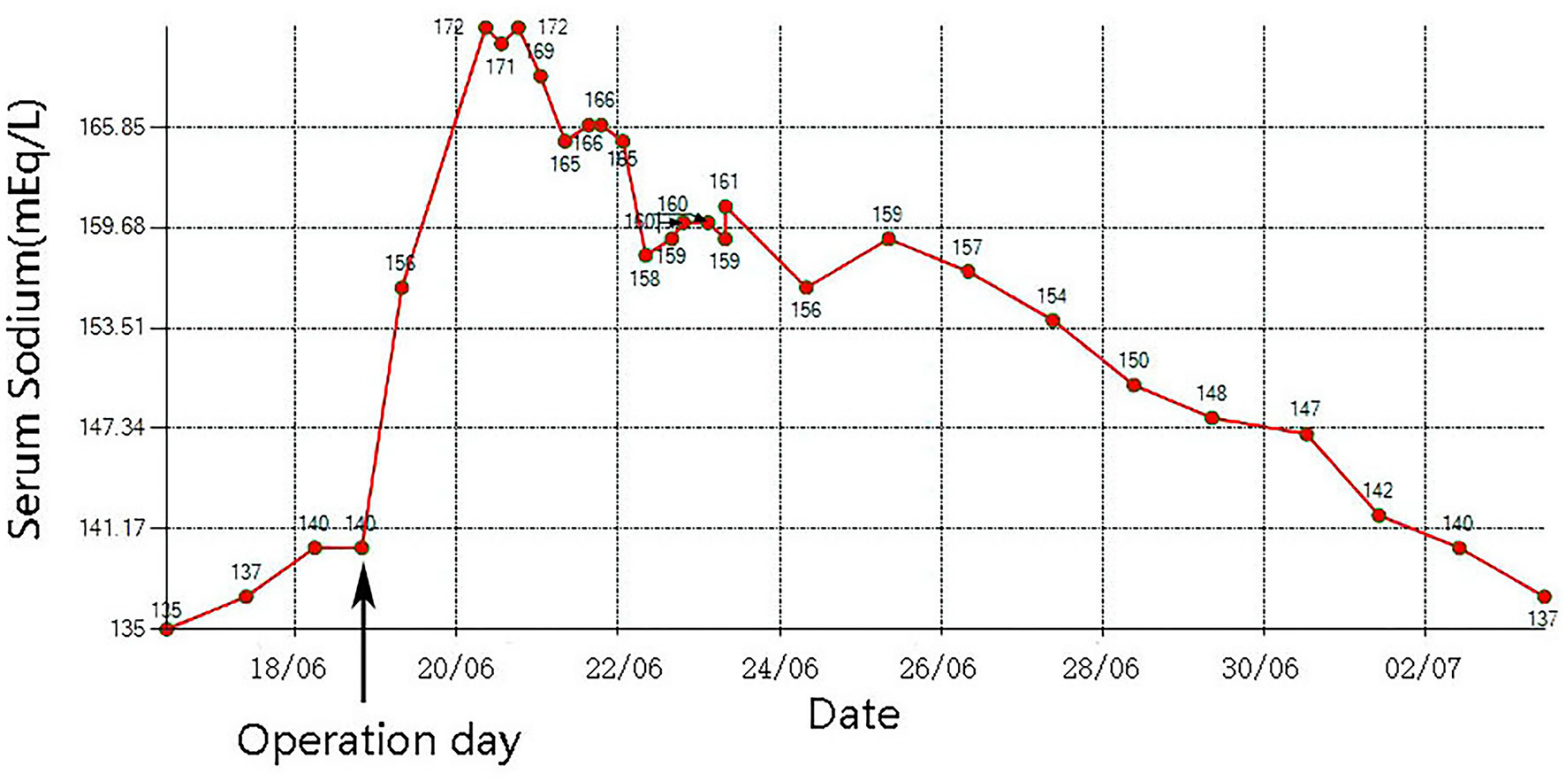
The change of serum sodium in this case.

**Figure 3:**
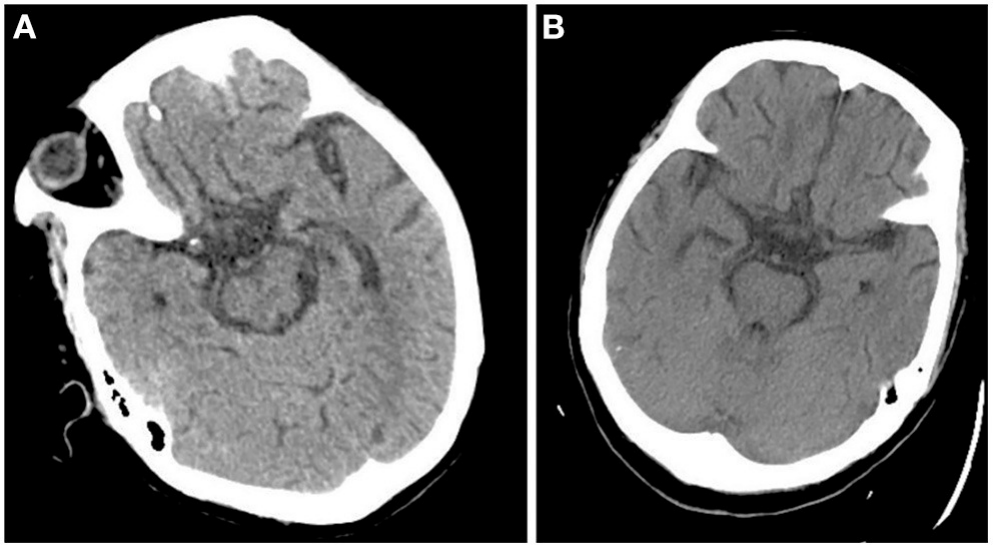
CT scan didn't demonstrate any hypodensity in pons 1 day after the operation **(A)** and 7 days after the operation **(B)**.

**Figure 4:**
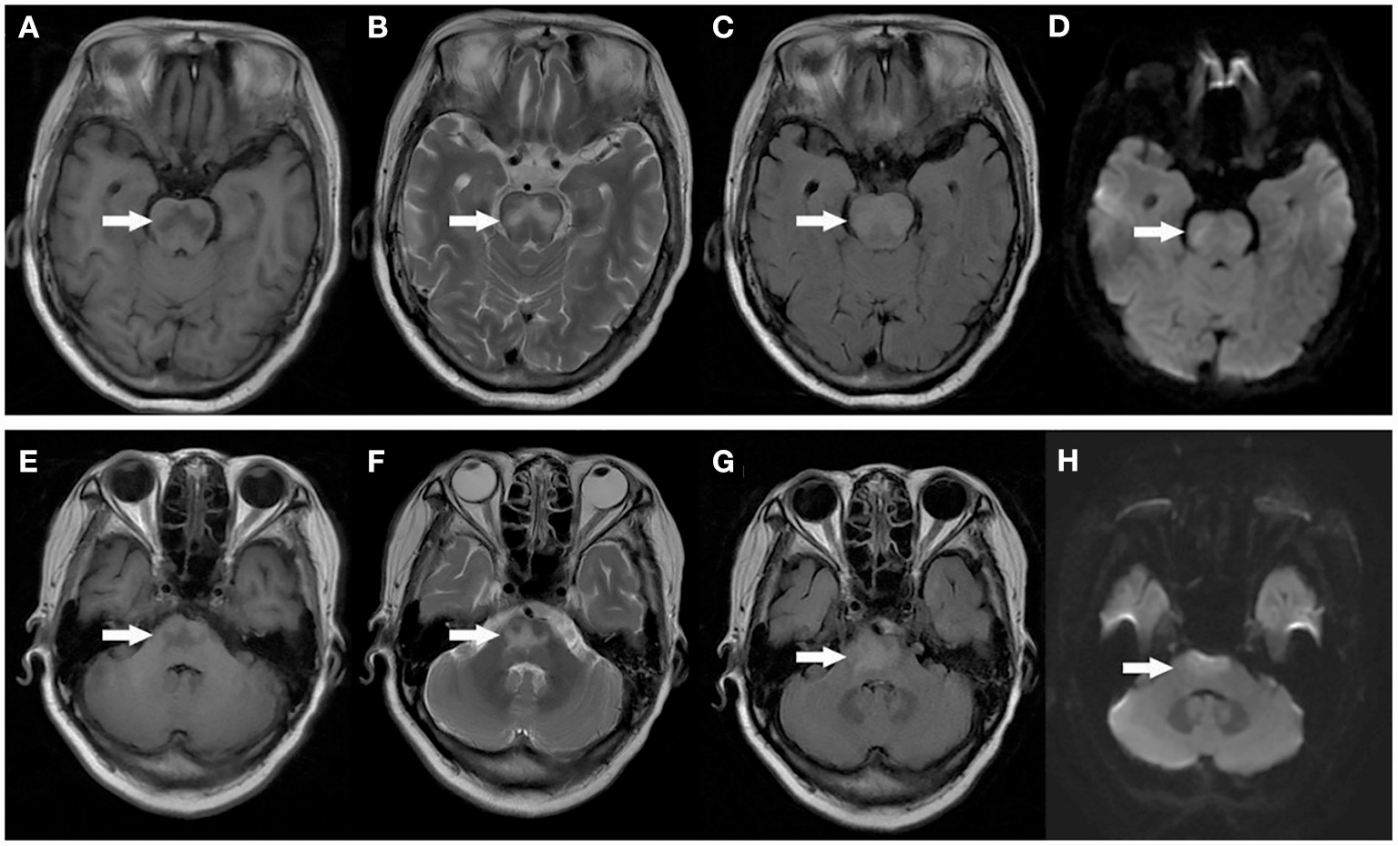
MRI image of the patient's head: Axial T1 weighted images demonstrating symmetric low signal in the central pons **(A,E)**. Axial T2 weighted images demonstrating symmetric high signal in the central pons **(B,F)**. T2 FLAIR images demonstrate symmetric high signal in the central pons **(C,G)**. DWI images didn't show any diffusion restriction in the central pons **(D,H)**.

After admission, the patient was prepared to be treated by TAVR. Before the operation, she received furosemide (20 mg p.o. qd, 1 day), spironolactone (20 mg p.o. qd, 5 days), torsemide (20 mg i.v. qd, 3 days; 20 mg i.v. bid, 2 days), and tolvaptan (7.5 mg p.o. qd, 2 days) for diuresis but showed little benefit. The patient's symptoms were worse, and her blood pressure dropped (SBP: 81–95 mmHg). Dobutamine was used immediately to maintain stable hemodynamics but failed. For unstable hemodynamics, she received emergency TAVR under the support of extracorporeal membrane oxygenation (ECMO) (venoarterial, pump rotation speed: 3,000 RPM, blood flow: 2.7 L/min). After the operation, vital signs were stable, and urine output increased markedly. On postoperative day 1, the patient's glomerular filtration rate was 45.2 ml/min, BUN was 15.45 mmol/L, creatinine was 85 μmol/L, serum osmolarity was 336 osmo/kg, urine osmolarity was 280 osmo/kg, urinary specific gravity was 1.009, and urine output was 5,720 ml/24 h (1,500 ml in the first 3 h after TAVR). Chest X-ray showed decreased pulmonary oedema and bilateral pleural effusion (Figure 1B), but serum sodium increased from 140 to 172 mmol/L (Figure 2). ECMO was discontinued on the first day after the operation. However, the patient's consciousness did not recover after the operation. CT brain scans were performed twice to look for lesions in her brain, but they did not reveal any hypodensity on CT images (Figure 3). Then, she underwent an MRI scan, and we found multiple lesions in the pons. We observed a symmetric low signal on T1-weighted imaging and a symmetric high signal on T2-weighted imaging and fluid attenuated inversion recovery (FLAIR) sequence imaging in the pons and medulla oblongata areas, which are called the trident sign (7) or Mercedes Benz sign (8). This classical radiological picture favors a change in ODS (Figure 4). However, a diffusion-weighted imaging (DWI) signal change was not observed in our case.

She received corrective treatment for hypernatremia (changing all drug solvents (if applicable) from normal saline to sterile water injection or glucose injection) and was given water through a nasogastric tube (dextrose in water, n.g. 20 ml/h; electrolyte-free water n.g. 20 ml/h), and her serum sodium returned to the baseline level. She received intravenous injection of human immunoglobulin 20 g daily for 5 days and supportive care. After treatment, she regained consciousness but had dysarthria and impaired muscle power. She was discharged 1 month later with dysarthria and a muscle power of grade 2 in the left limbs and grade 5 in the right limbs. Two months later, we phoned the patient, and she told us that her phonetic function and muscle power had returned to normal.

## Discussion

Osmotic demyelination syndrome (ODS), which is characterized by widespread degeneration of myelin within the central nervous system (CNS), was first described by Adams et al. (9). Nearly two decades (mid-1970s) after it was described, people found that its common cause is rapid overcorrection of chronic hyponatremia. The increase in serum sodium causes an increase in serum osmolality. An increase in serum osmolality causes increased osmolality in the brain, which causes shrinkage of astrocytes and oligodendrocytes, resulting in their apoptosis and inflammation and disruption of the blood–brain barrier and thus leading to demyelination. However, in this case, the patient's serum sodium was normal when she was admitted to the hospital. After admission, she was diagnosed with severe aortic stenosis and resulting pulmonary oedema. To relieve the patient's symptoms, a massive dose of diuretic was used, but the patient's urinary output did not increase, and her symptoms worsened. In the past, patients in this situation were very difficult to manage. Fortunately, the development of TAVR and ECMO has afforded patients another chance for cure. The use of VA-ECMO as a salvage therapy in cardiogenic shock is becoming a current practice (10). The patient's hemodynamics were stable after ECMO was performed, and she underwent emergency TAVR. After the operation, the patient's urinary output increased prominently (1,500 ml in the first 3 h after TAVR), and serum sodium increased from 140 to 156 mmol/L and then to 172 mmol/L. A variety of therapies were used immediately to decrease serum sodium quickly, but the effect was not satisfactory (Figure 2). The patient's consciousness changed after the operation, and clinical evidence indicated that she had ODS. This case shows that ODS occurs not only in patients with chronic hyponatremia but also in patients with normal serum sodium, and that rapid sodium increases should be considered in these patients.

The frequency of symptoms and signs of heart failure (such as dyspnoea and pulmonary oedema) among patients with aortic stenosis varies depending on the stage of disease. According to the REMEDY study, the incidence of heart failure in patients with rheumatic valvular heart disease is 33.4% (11). Diuretics are often used in these patients to relieve symptoms in current clinical practice. With the development of the operation, TAVR has become a very useful therapy for patients with severe aortic stenosis. Due to blood flow disturbance from the left ventricle to the aorta in patients with severe aortic stenosis, acute prerenal kidney failure is likely caused by low kidney perfusion pressure. When aortic stenosis is relieved by TAVR, renal perfusion will improve, and renal blood flow will increase within a short time. Combined with the residual effect of a massive dose of diuretic used before surgery, the patient's urinary output will increase very quickly after the operation, and serum sodium will increase markedly, which is an important issue warranting attention. Balances of fluid volume and electrolyte levels in this phase are quite fragile. Although closely monitored and carefully managed in this case, due to systemic factors, these balances are still difficult to manage.

ODS symptoms vary, including lethargy, quadriparesis, dysarthria, ophthalmoplegia, ataxia, and even coma or death and depend on the degree of pontine involvement and the presence of extrapontine lesions (12, 13). Hildur Aegisdottir et al. analyzed 83 patients with ODS and found that 73 patients (88%) had bulbar symptoms, while 71 patients (85.5%) had dysarthria/dysphagia. Furthermore, 65 (78.3%) had limb paresis at diagnosis, and 10 (12.0%) were in a locked-in state (14). In this case, the patient's main symptoms were coma, dysarthria, and impaired muscle power, indicating injuries in the nervous system. The rate of clinical neurological events after TAVR ranges from 3 to 7% (15, 16). Patients undergoing TAVR are at an increased risk for developing acute cerebral hypoperfusion during balloon aortic-valvuloplasty/valve deployment (17). Diffusion-weighted magnetic resonance imaging (DWI) revealed new cerebral DWI lesions among >70% of patients after TAVR, regardless of the valve type or implantation strategy (18–20). In this case, when we found that the patient's consciousness had changed after TAVR, stroke was first considered. However, two CT scans did not demonstrate any hypodensity on postoperative day 1 and postoperative day 2, which did not favor stroke. After MRI was performed, a classical radiological picture was evident in the pons, which favors a change in ODS.

Early imaging evidence of changes in ODS may not appear on CT. Patients suspected of having ODS should undergo brain MRI. The typical MRI findings of ODS are a symmetric low signal on T1-weighted images and a symmetric high signal on T2-weighted and FLAIR images in the central pons or associated extrapontine structures (21, 22). The classical radiological picture is called the trident sign, butterfly sign, or Mercedes Benz sign (8). The sensitivity of DWI is still controversial. Kimberly A. Ruzek et al. found that restricted diffusion is the first imaging manifestation of CPM, which occurs within 24 h of the clinical onset of tetraplegia and before detection of abnormalities on conventional MRI images (23). However, Förster et al. analyzed eight ODS patients and found that DWI changes did not regularly precede tissue changes detectable on conventional MRI sequences (24). Therefore, Johann Lambeck believed that DWI, T1, T2, and T2 FLAIR sequences were equivalent for detection purposes (2). These imaging changes may not be clearly visible until 1–2 weeks later (25). In our case, MRI showed a symmetric low signal on T1-weighted and a symmetric high signal on T2-weighted and FLAIR imaging in the pons and medulla oblongata areas. DWI signal changes did not occur in our case.

Apart from supportive therapy and experimental therapies, no definitive treatment has been established for ODS. Ludwig et al. reported 2 cases of ODS in which treatment with plasmapheresis and intravenous immune globulin improved long-term neurologic outcomes (26). Kengne et al. found that treatment with dexamethasone resulted in fewer neurological manifestations 24 h after correction of chronic hyponatremia in a rat model of ODS, but dexamethasone failed to reduce mortality at 5 days (27). Chemaly et al. reported a case of a 13-year-old girl diagnosed with ODS who received 0.6 mg i.v. of thyrotropin-releasing hormone (TRH) daily for 6 weeks until complete recovery (28). Haruyuki Suzukican et al. found that minocycline could protect against ODS by inhibiting the activation and accumulation of microglia at the site of demyelinative lesions in a rat model of ODS. Recently, Wijayabandara et al. reported a case in which plasmapheresis may remain effective in reversing ODS several weeks after the initial osmotic insult (29). To our knowledge, the small amount of evidence on the treatment of ODS is based purely on case reports, animal experiments or small case series (2). The treatment of patients with ODS requires further study. However, depending on disease severity, these therapeutic choices should be considered in the clinical management of ODS.

## Conclusion

In summary, to our knowledge, this is the first reported case of ODS after TAVR. ODS occurs not only in patients with rapid overcorrection of chronic hyponatremia but also in patients with normal sodium. With the development of the operation, TAVR has become a very useful therapy for patients with severe aortic stenosis. After a patient receives TAVR, kidney perfusion pressure quickly returns to normal, and with the residual effect of a high-dose diuretic administered before surgery, balances of fluid volume and electrolyte levels in this phase are quite fragile and must be carefully managed. If a patient has neurological symptoms/signs in this phase, ODS should be considered, and MRI might be necessary.

## Data Availability Statement

The original contributions presented in the study are included in the article/supplementary material, further inquiries can be directed to the corresponding author/s.

## Ethics Statement

Ethical review and approval was not required for the study on human participants in accordance with the local legislation and institutional requirements. The patients/participants provided their written informed consent to participate in this study. Written informed consent was obtained from the individual(s) for the publication of any potentially identifiable images or data included in this article.

## Author Contributions

XJ wrote the first draft of the manuscript and revised it. YW was involved in the conception and design of the work and revised the manuscript. All authors contributed to the article and approved the submitted version.

## Conflict of Interest

The authors declare that the research was conducted in the absence of any commercial or financial relationships that could be construed as a potential conflict of interest.

## Publisher's Note

All claims expressed in this article are solely those of the authors and do not necessarily represent those of their affiliated organizations, or those of the publisher, the editors and the reviewers. Any product that may be evaluated in this article, or claim that may be made by its manufacturer, is not guaranteed or endorsed by the publisher.
